# Circulation of the Blood

**Published:** 1886-06

**Authors:** 


### CIRCULATION OF THE BLOOD.

To Michael Servetus is due the credit for the discovery of the circulation of the blood through the lungs, from the right to the left side of the heart. His description, complete as it was, was merely incidental to a theory on the nature of the soul, which was regarded as so heretical that, by the instigation of John Calvin, the illustrious discoverer was burned alive at the stake at Geneva in the same year, 1553. A copy of the book of Servetus is preserved in the library of the French Institute. Cesalpinus, of Pisa, Italy, was the first to use the expression, "circulation of the blood," and to observe that on compressing a vein the swelling is always below the point of obstruction. To Fabricius is credited the discovery of the valves in the veins, although Etienne, 1545, Amatus Sussitanus, 1551, and Eustachius—after whom the Eustachian tube is named—1563, had described valves in different vessels. Piccolhominus, 1586, gave a clear account of the valves of the veins; but Fabricius, 1603, published the fullest and most accurate description, and demonstrated their existence to Harvey, in Padua. Turning his attention to the subject, Harvey added greatly to the few isolated facts then known by studying the exposed heart in living animals. By experiments upon fishes and serpents he proved that the heart receives the blood from the veins and discharges it into the arteries. He applied a ligature to the veins, which had the effect of cutting off the supply to the heart so that it became pale and flaccid, and by removing the ligature the blood could be seen flowing into the organ. On the contrary, when the artery was ligated the heart became distended, and continued to as long as the obstruction remained. Harvey lost his medical practice by his scientific discoveries, and declared he could not get a physician over forty years of age to believe him.

